# Increased spatial resolution of sampling in the Carpathian basin helps to understand the phylogeny of central European stream-dwelling gudgeons

**DOI:** 10.1186/s40850-021-00069-7

**Published:** 2021-03-06

**Authors:** Péter Takács, Árpád Ferincz, István Imecs, Balázs Kovács, András Attila Nagy, Katalin Ihász, Zoltán Vitál, Eszter Csoma

**Affiliations:** 1 Balaton Limnological Institute, Centre for Ecological Research, Klebelsberg Kuno str. 3, Tihany, H-8237 Hungary; 2 Department of Freshwater Fish Ecology, Institute of Aquaculture and Environmental Safety, Hungarian University of Agriculture and Life Sciences, Páter Károly str. 1, Gödöllő, H-2103 Hungary; 3 ACCENT GeoEcological Organization, str. Ciucaș nr. 62/A, 535100 Băile Tușnad, Romania; 4 “MilvusGroup” Birdland Nature Protection Association, Crinului nr 22, 540343 Tîrgu Mureș, Romania; 5 Evolutionary Ecology Group, Hungarian Department of Biology and Ecology, Faculty of Biology and Geology, University Babeş-Bolyai, Strada Clinicilor 5–7, 400006 Cluj-Napoca, Romania; 6 National Agricultural Research and Innovation Centre, Research Institute for Fisheries and Aquaculture, Anna-liget u. 35, Szarvas, 5540 Hungary; 7 Department of Medical Microbiology, Faculty of Medicine, University of Debrecen, Nagyerdei krt. 98, Debrecen, H-4032 Hungary

**Keywords:** Carpathian basin, Isolation, Speciation, Cyprinidae, *Gobio*

## Abstract

**Background:**

Phylogenetic studies of widespread European fish species often do not completely cover their entire distribution area, and some areas are often excluded from analyses than others. For example, Carpathian stocks are often omitted from these surveys or are under-represented in the samples. However, this area served as an extra-Mediterranean refugia for many species; therefore, it is assumed that fish stocks here may show special phylogenetic features. For this reason, increased spatial resolution of sampling, namely revealing genetic information from unexamined Carpathian areas within the range of doubtful taxa, may help us better understand their phylogenetic features. To test this hypothesis, a phylogenetic investigation using a partial mtCR sequence data was conducted on 56 stream-dwelling freshwater fish (*Gobio* spp.) individuals collected from 11 rivers of the data-deficient Southeastern Carpathian area. Moreover, we revieved the available phylogenetic data of Middle-Danubian stream-dwelling gudgeon lineages to delineate their distribution in the area.

**Results:**

Seven out of the nine detected haplotypes were newly described, suggesting the studied area hosts distinct and diverse *Gobio* stocks. Two valid species (*G. obtusirostris, G. gobio)*, and a haplogroup with doubtful phylogenetic position” G. sp. 1" were detected in the area, showing a specific spatial distribution pattern. The distribution of the detected lineages in the Middle-Danubian area correspond with recent and paleo hydrogeographic features, at the same time mainly on their bordering areas show considerable overlap.

**Conclusions:**

Despite the relatively limited geographic range of the study, our results provide important information which contributes to a better understanding of the phylogenetic, taxonomic and distribution features of Central European gudgeons. The genetically confirmed distribution data of the indicated lineages corresponds well with the recent and near-recent hydrogeographic features of the area, and assumes several hybrid zones in the Carpathian Basin. Additionally, the results show that the middle and lower Danubian watershed cannot be excluded from the range of *G. gobio*. Moreover, the” G. sp. 1", is slightly differentiated but phylogenetically distinct entity, and is the only *Gobio* taxa thus far detected in the middle and lower Tisza-basin. However, further investigations are necessary to clarify the taxonomic position of this group.

## Background

Molecular genetic methods have become the basic tools for phylogenetics and taxonomy in the last two decades [1, 2]. These methods are also widely used both for supra- and intraspecific studies [3, 4]. Moreover, their sensitivity makes them suitable for elucidating the phylogenetic and taxonomic relationships of those “complicated” European fish taxa (e.g. barbels, European minnows, loaches, spirlins, bitterling, grayling, etc.) that have not been revealed successfully by conventional (i.e. phenotype based) methods [5–11]. Results of these studies show that many hitherto widespread species have to be separated into a number distinct entities by their phylogenetic features. Moreover, as the result of these genetic studies the taxonomy of these groups has been significantly changed, and/or new species have also been described from smaller catchments [12, 13].

Concurrently, phylogenetic investigations are costly and time consuming, and have substantial consumable requirements [14]. For this reason, only a limited number of samples are typically analyzed. In the case of wide-ranging species the authors generally took into account the glaciations caused along the North-South genetic diversity gradient [15] and often gave special attention to the Southern areas of Europe (e.g. Balkan peninsula). At the same time some other larger areas, even entire river basins, have been excluded from these analyses. In many cases it is striking that the inner and Southeastern areas of the Carpathian basin are underrepresented and/or completely excluded from the regional surveys [6, 9, 16, 17]. Although the studied species (e.g. gudgeons, stone loach, bitterling, European minnow) occur and some are even considered to be particularly common in the above mentioned areas [18, 19]. Moreover, it has been shown that the Carpathian basin,since as it never glaciated during the ice ages, served as an extra-Mediterranean refugia and source of recolonisation to Northern European areas for many plant and animal groups [20, 21]. Therefore, surveys undertaken on Carpathian stocks may help considerably to better understandthe phylogeny of the studied taxa. In our present work, using stream-dwelling gudgeons (*Gobio* spp.) as an example, we demonstratehow a genetic survey made on their stocks collected from a relatively small Carpathian area can help us to understand the still-confusing phylogenetics and taxonomy of a commonly-distributed higher taxon.

Gudgeons (Gobionidae) are among several commonly-distributed groups in the European fish fauna whose phylogenetic and taxonomic relationships has not yet been clarified in detail [22, 23]. This ancient and distinct cypriniform family [24] consists of 30 genera and 130 species. The family is widely distributed throughout Eurasia, from Spain east to Japan and south to central Vietnam, and appear in highest numbers in eastern Eurasia [25–28]. These small-sized (~ 10 cm SL) and short-lived fish show great ecological, and morphological variation [25, 26, 29]. Some of them are benthic and rheophilic, while others are semi-pelagic. Many of them can be found in hilly streams to middle-sized rivers, while others appear in tropical swamps [28]. Gudgeons are known to be commonly distributed in Middle Europe and in the Carpathian basin [18, 30]. Results of our recent countrywide study showed that stream-dwelling gudgeons (*Gobio* spp.) were detected in 34% of the 767 Hungarian river sections that were surveyed, and that they characteristically occupy hilly streams and rivers of the inner area of the Carpathian Basin [19].

The taxonomy of this ancient cypriniform family is still unclear, with several gudgeon taxa/species having a controversial taxonomic position [31, 32]. Until the end of the twentieth century, the eponymous species of the family, the European gudgeon [*Gobio gobio* (Linnaeus, 1758)], was accepted as the only, but widely-ranged superspecies in western Eurasia. However, its 19 subspecies have been described in the larger hydrographic regions. Rheophilic, limnophylic and intermediate forms have also been reported from some regions [29]. Using traditional taxomic methods, altogether three *G. gobio* subspecies have been noted so far from the waters of the Carpathian basin. *Gobio gobio obtusirostris* Valenciennes, 1842 has been detected in the western (Danubian) area of the basin, and two drainage systems of the largest Danubian tributary, the River Tisza (L = 962 km, A = 157,000 km^2^). One of the latter two subspecies, *G. gobio carpathicus* Vladykov, 1925 [33] has been reported in the northeast area of the Tisza drainage, while *G. gobio muresius* Jászfalusi, 1951 has been described in the Southeastern Carpathian area, in the upper section of the River Maros/Mures (L = 749 km, A = 27,049 km^2^) which receives waters of the Southern Transylvanian region and carries them into the Tisza River [34]. The taxonomic position of this latter subspecies is quite controversial. Bӑnӑrescu [35] designated it as the rheophilic form of *G. gobio obtusirostris*, and in his other publication he stated that this group does not have a clearly defined distribution area, therefore it is” far from being a valid subspecies” [29].

**Fig. 1:**
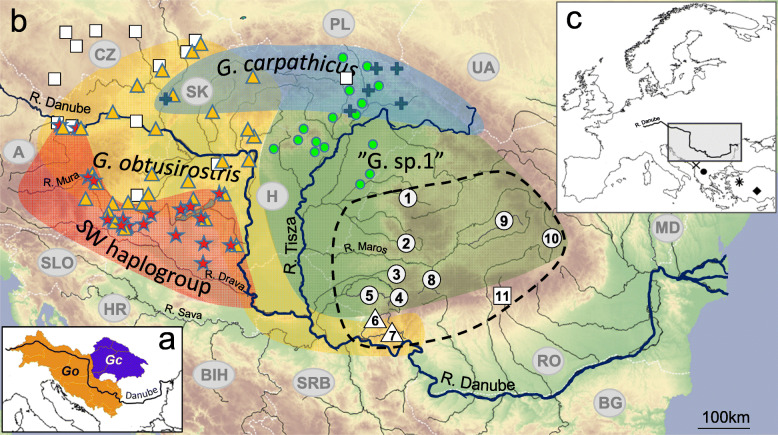
The distribution of the recently accepted Middle Danubian/Carpathian *Gobio* species (**a**) and the estimated distribution of *Gobio* lineages in the Carpathian basin and its surrounding areas derived from mtCR phylogenetic data (**b**). On subfigure a: orange: *G. obtusirostris* (Go), blue: *G. carpathicus* (Gc). The different shaped signs at sample sites show the appearence of different haplogroups: *G. obtusirostris*: orange triangle*,* SW haplogroup: red star, *G. carpathicus*: blue cross, *G. gobio*: white rectangle, G. sp.1: green circle. Yellow, red, blue and green shaded areas show the estimated distribution area of *G. obtusirostris*, the Southwest haplogoup, *G. carpathicus* and the G. sp. 1 haplogroups, respectively. The geographic distribution of our 11 sample sites in the Southeast area of Carpathian basin is shown by numbered white icons. Site numbering corresponds with Table 1. A black dashed line shows the border of the newly surveyed area. White dotted lines show country borders. Country codes are showed by grey letters. Location of the studied area in Europe is indicated on subfigure **c**. In this subfigure the collection sites of other close relative *Gobio* species also used for the phylogenetic analyses are indicated: *G. skadarensis*: ×, *G. ohridanus*: ●, G. sp.2: *, *G. insuyanus*: ♦. Base map was generated by QGIS sofware using layers freely available from European Environment Agency (EEA). Sources of distribution data on subfigure a: https://www.iucnredlist.org/species/135651/4171068, and: https://www.iucnredlist.org/species/135501/4133848. The certain localities and the presumed distribution areas of the *Gobio* lineages indicated from the Middle Danubian area were drawn using the information provided by Mendel et al. [17], Takács et al. [37], Zangl et al. [38]. Note: due to the relatively small scale of the map the sample sites are only approximate positions, and if more lineages appeared at the same sites the icons were slightly shifted

The first results of genetic investigations basically confirmed the suppositions based on traditional taxonomic methods. However, despite the generally low interspecies genetic differences, several *Gobio* subspecies have been raised to species level [17, 36]. Moreover, the distribution area of certain species has been considerably changed. For instance, the Middle Danubian watershed – including the Carpathian basin – has been excluded from the area of *G. gobio.* Concurrently, two of its subspecies have been raised to species level. From the western drainage area, the Danubian gudgeon - *G. obtusirostris*, and from the Tisza drainage the Carpathian gudgeon - *G. carpathicus* have now been recognized as valid species [22] (see: Fig. 1a). Mendel et al. [17] provided additional information about this area. They included *G. carpathicus* in the Dyje river from the Czech Republic as well, therefore its distribution is not limited solely to the Tisza drainage. At the same time, in addition to *G. carpathicus*, another genetically very different entity was reported from the Tisza drainage. The „Gobio sp.1″ (sic!) is called as a “species-in-waiting”.

The recently published finer-scaled studies, focusing on the *Gobios* of the Middle Danubian hydrosystem, considerably contributed to our knowledge of the distribution and phylogeny of this species group. Thus, the haplotype of *G. gobio* has been indicated in the inner area of the Carpathian basin, but similarly to *G. carpathicus*, and this species seems to be sporadic in the streams of the inner area of the basin. At the same time *G. obtusirostris* has been shown to be the dominant gudgeon species in the NW region of the Carpathian basin [37]. On the other hand, in the SW area of the basin, and in the middle region of the River Tisza drainage system (eastern part of the basin), two allopatric “cryptic entities” are the most frequent *Gobio* taxa. The „G. sp.1″ is strictly located in the Middle Tisza drainage, and this is the dominant stream-dwelling gudgeon. Phylogenetically, „G. sp.1″ occupies an intermediate position between *G. obtusirostris* and *G. gobio.* Additionally, a hitherto unknown haplogroup, called „SW haplogroup” or „Balcanic clade”, has been detected in the Mura-Drava system. Phylogenetic analyses showed that this new group should be classified between *G. obtusirostris* and “G. sp.1″; however, it shows higher similarity with *G. obtusirostris* [37, 38]. The above-mentioned information suggests that the phylogenetic patterns of the stream-dwelling gudgeons living in the Carpathian basin have not been studied extensively. Therefore, some new groups/lineages might be present in those sub-basins that have not been previously investigated.

Considering the hydrography of the Carpathian basin, the hydrosystem of the previously unexamined Southeast area was chosen for investigation. Since the investigated area is located between the already examined Southwestern (Mura-Drava) and central Tisza sub-basins, if isolation by distance is assumed in this area, any haplotypes that appear in this area would fill the phylogenetic gap between” G. sp.1″ and the „SW haplogroup”. Our assumption is based on the fact that this area of the Carpathian basin is especially rich endemic plant and animal species [39–42]. In our opinion, by revealing phylogenetic information on the stocks of this unexamined area, the geographic range of valid *Gobio* species could be specified and clarified. Moreover, their interspecific genetic distances may also be modified. Additionally, the results of the phylogenetic studies on gudgeons collected in the Southwest region of the Carpathian basin may answer questions of whether separated, phylogenetically distinct *Gobio* species can be found in this area; or a „quasi genetic continuum” exists, formed by genetically less-distinct clusters living in the larger subdrainages of the Carpathian basin. Therefore, the aims of our study were: 1) to provide phylogenetic information about the characteristic fish species of a hitherto data-deficient area, 2) to clarify the phylogenetic relations of *Gobio* stocks inhabiting the inner area of the Carpathian basin. We also reviewed and compared the recently accepted distributions and the phylogeneticaly-verified *Gobio* distribution data in the Carpathian basin and its surrounding catchments.

## Results

Sequencing resulted in a 612 bp sequence of CR mtDNA alignment that contained 25 polymorphic sites, with no gaps or missing data, and the 56 analysed samples collapsed to 9 haplotypes (H1-H9). Seven out of these nine haplotypes have previously been unknown and have been deposited in the GenBank database under accession N^**o**^s. MT547546–52. Based on BLASTn analyses, H2 and H3 haplotypes (acc. N^**o**^s: MT967499–500) showed a complete match with the haplotypes KC757339 and KC757341 in the GenBank, respectively. The newly-indicated haplotypes from the study area were compared to haplotypes of gudgeon species and lineages described from adjacent regions (e.g. Central Europe, Balkan Peninsula, and Anatolia). Indels appeared when relatively distant *Gobio* relatives were involved in the analyses. Alignment of our sequences with *G. insulyanus* haplotypes revealed a deletion at the position 100; similarly there is a deletion in the case of *G. insuyanus* and *G. ohridanus* haplotypes at the position 336. If *R. vladykovy* is included in the analysis two more insertions and deletions appeared at the positions 224, 411, and 417, 418 respectively. Pairwise genetic differences (uncorrected p-distances) between the newly-indicated nine haplotypes ranged 0.02–0.33 concerning substitutions. These values ranged between 0.00 and 0.105 for the entire haplotype pool (including literature data, and outgroup haplotype also). Among the seven new haplotypes revealed in this study, haplotype H1 showed the highest similarity to the *G. gobio* species, the haplotypes H2-H6 proved to be members of the” G. sp.1″ group, while the haplotypes H7-H9 belong to *G. obtusirostris*. Therefore, from the 56 phylogeneticaly identified individuals, 5, 41, and 11 proved to be *G. gobio,* G. sp.1, and *G. obtusirostris* respectively (Table 1)*.* Intragroup nucleotide differences ranged between 0.2–0.3% (mean ± SD = 0.29 ± 0.13%) in the case of *G. obtusirostris* (including haplotypes H7-H9) and between 0.2–0.5% (mean ± SD = 0.22 ± 0.09%) in the case of the” G. sp.1″ group (H2-H6). The mean of haplogroup nucleotide differences were 1.19 ± 0.19% and 2.4 ± 0.2% between G. sp.1 and *G. Gobio*, and between G. sp.1 and *G. obtusirostris*, respectively. The mean ± SD difference between *G. obtusirostris* and *G. gobio* haplotypes was 3.1 ± 0.16%. The distribution of the three haplogroups were clearly separated from each other, and no site with phylogenetically mixed assemblages was found in our study.

**Table 1:** Data of the sampled river sections

N^**o**^	River name	Sample site	Date	Geo-coordinates	Elev. (m)	Haplotype frequencies									Σ
						H1	H2	H3	H4	H5	H6	H7	H8	H9	
1.	Crișul Repede/Sebes-Körös	Bratca/Barátka	2016.09.06	46.933, 22.664	354		6								6
2.	Crișul Alb/Fehér-Körös	Leasa/Sövényes	2016.09.06	46.277, 22.535	209		2								2
3.	Bega /Béga/	Făget/Facsád	2018.09.26	45.846, 22.131	139						4				4
4.	Timiș/Temes	Zăgujeni/ Zaguzsén	2018.09.25	45.478, 22.180	178					3	3				6
5.	Bârzava/Berzava	Gătaia/ Gáttája	2018.09.27	45.441, 21.454	109						6				6
6.	Caraș/ Krassó	Grădinari/Kákófalva	2018.09.27	45.109, 21.581	102							4	1		5
7.	Nera Néra	Bozovici/Bozovics	2018.09.27	44.902, 21.988	235									5	5
8.	Strei/ Sztrigy	Petreni/Petrény	2018.09.28	45.786, 23.015	208			5							5
9.	Târnava Mică/Kis-Küküllő	Chibed/Kibéd	2016.09.09	46.539, 24.983	382			5	1						6
10.	Olt/Olt	Miercurea Ciuc /Csíkszereda	2016.09.09	46.319, 25.828	652		5	1							6
11.	Argeș/Argyas	Rotunda	2016.09.07	45.268, 24.656	525	5									5
					Σ	5	13	11	1	3	13	4	1	5	**56**

**Fig. 2:**
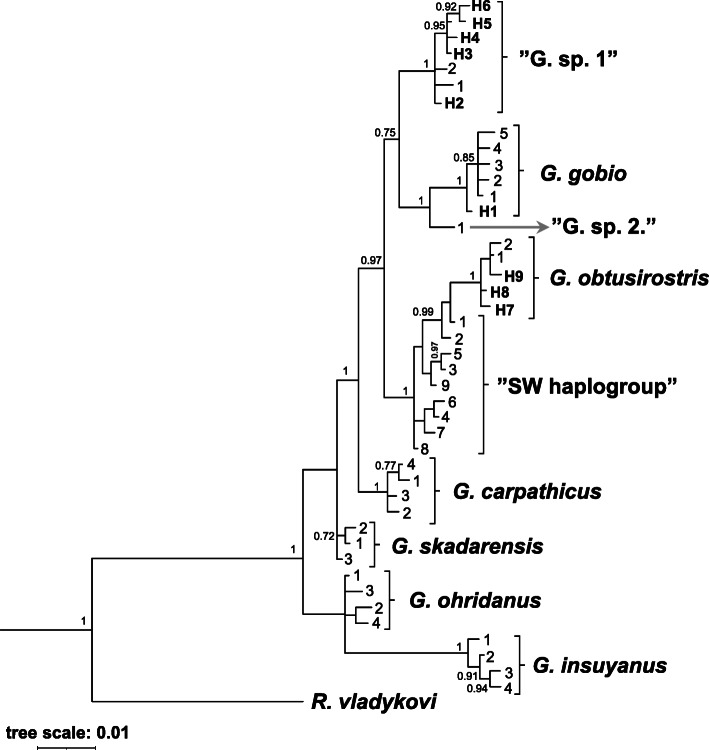
Bayesian inference phylogram of the genus *Gobio* based on mtCR sequences of the investigated *Gobio* individuals and literature data. Posterior probabilities (> 0.7) are listed near the nodes. The names of valid species are italicized; the name of the three groups with an uncertain taxonomic position is enclosed in quotation marks. Haplotypes revealed in this study are marked with their codes (H1-H9) (for more detalis see Tables 1 and 2). A tree scale of 0.01 corresponds to inferred evolutionary changes. Details of the DNAsequences retrieved from GenBank are shown in Table 2

**Fig. 3:**
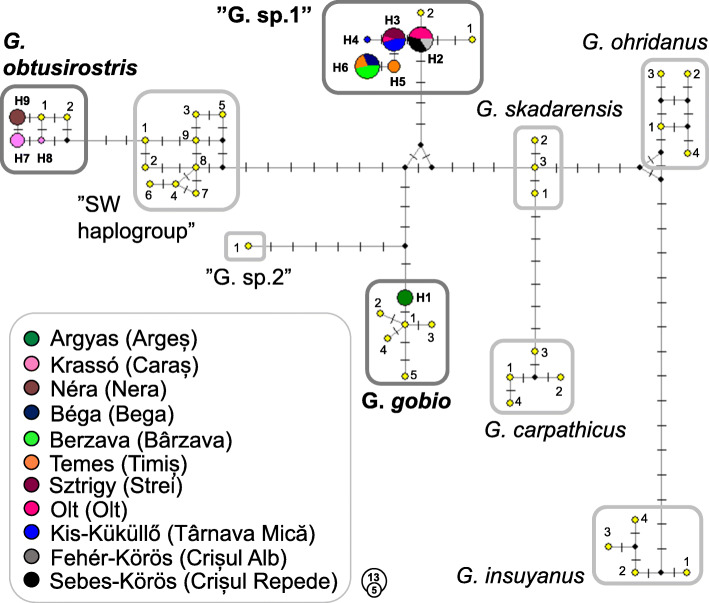
Median-Joining network of mtCR sequence data of the investigated 56 Gobio individuals and close relative *Gobio* haplotypes. Circle size is relative to the number of individuals carrying the same haplotype. Color codes show the origin of the individuals sharing the same hapylotypes. Line length refers to the genetic distances of haplotypes. Each vertical line is one mutation step. Small black circles represent median vectors (missing or theoretical haplotypes). H1–H9: Haplotypes of the 56 specimens analysed in this study. Previously published haplotypes are marked by yellow circles. The numbers in each box correspond with the numbers displayed in Table 2. The names of valid species are italicized; the name of the two groups with an uncertain taxonomic position is enclosed in quotation marks. The haplogroups indicated during this study are highlighted with a dark gray frame and with bold letter type

Code, Romanian and Hungarian names of the sampled rivers and sampling sites, collection date, and geo-coordinates, altitude above sea level (Elev.) and haplotype frequencies in each sampling sites. (H1-H9 indicated haplotypes during this study). Site numbers and haplotype codes correspond with Figs. 1, 2 and 3. Previously unknown haplotypes are highlighted with bold letter type

Bayesian inference tree and Network (Figs. 2 and 3.) showed the newly-revealed haplotypes fit well with known haplotypes. This result was reinforced by the findings of the Blast analyses; thus, only previously-identified lineages are present in the area. Network analysis and the nucleotide difference computations showed that „G. sp.1″ displays a similar degree of separation from the two closely-related valid species (*G. obtusirostris*, and *G. gobio*). Moreover, both analyses showed that the phylogenetically doubtful groups (G. sp.1, G. sp. 2, and SW haplogroup) blur the differences between valid species.

The available phylogenetically-verified *Gobio* distribution data [17, 37, 38] from the region is presented with our results in Fig. 1. Results show that the distribution pattern of different Middle-Danubian *Gobio* lineages much more complex than the currently accepted pattern (Fig. 1 a, b). The Danube gudgeon was genetically-verified as occupying the western part of the basin, and along the Danube, but there was no data to confirm its presence in the Sava system. The phylogeneticaly-confirmed *G. carpathicus* localities are limited only to the Northern areas of the Carpathian Basin, and to a much smaller area than was previously accepted (see: Fig. 1a,b). The” G. sp. 1″ lineage can be considered to be dominant in the eastern and southeastern part of the basin. The indicated lineages are characterised by a pronounced hydrogeographic pattern, especially at the boundaries of their distribution areas, and they often appear in the same sampling sites. These contact zones can be observed in the western part of the Carpathian Basin between the SW haplotype and *G. obtusirostris.* On the NW part of the middle Danubian area *G. obtusirostris* has contact zones with *G. gobio* and even presumably with *G. carpathicus*. The distribution patterns also show a significant overlap between *G. carpathicus* and” G. sp.1.” in the Northern Carpathians. However, we found no evidence that „G. sp. 1″ would occupy an overlapping area with either *G. obtusirostris* or the SW haplogroup.

## Discussion

Phylogenetic data currently available on Carpathian populations of commonly-distributed fish species (e.g. pike, spirlin, loaches and minnows) show significant genetic differences from those living in Western European waters [e.g. [12, 13, 43]]. These results suggest that studies conducted on Carpathian populations may significantly expand our knowledge about the phylogeny of other species and species complexes as well. Moreover, the recent note of Krizek et al. [10] highlights that relevant taxonomic and phylogenetic findings can also be made by analyzing a limited number of individuals, if they are collected from the appropriate region, and if the newly-revealed phylogenetic information is analysed together with appropriately-selected data from the literature. In our case, despite the limited geographic range (~ 50′000 km^2^) of the studied area, and the relatively low sample size, the low within-site genetic variability and the geographic distribution of the indicated haplotypes suggests that the results of our investigation provides reliable and relevant information about the phylogenetic features of gudgeons living in the study area.

Altogether, nine haplotypes classified into two valid species and a haplogroup with doubtful phylogenetic and taxonomic position were found in our study. Seven of the revealed nine haplotypes were newly described, and these data considerably expand our knowledge on the genetic diversity of Carpathian Gobio stocks. The hydrogeographic distribution of the shown haplogroups/species shows an interesting pattern as well. A hitherto unknown haplotype of *G. gobio* was only observed in the upstream section of the River Argyas at Rotunda village, about 300 km from the Danubian estuary. Considering our recent knowledge about this species [44], this result seems to be an important data point in its distribution; namely because it confirms previous notes [17] showing the sporadic presence of *G. gobio* in the middle and lower Danubian watershed.

Similarly to the middle catchment of the River Tisza drainage [37], we did not detect the presence of haplotypes of the Carpathian gudgeon from the southeast area of the Carpathian Basin either. Therefore it seems that its range is much narrower than previously known [45] and it is limited solely to the northern Carpathian areas. Altogether, three haplotypes were found in the River Néra and R. Krassó (Sites 6 and 7), and all of them was proved to be new, hitherto unknown haplotypes of the Danubian gudgeon (*G. obtusirostris*). Therefore, our results clearly show that this species is present in the Southern Carpathian area, but only in these two direct Danubian inflows. Interestingly, in the R. Temes, which also flows directly into the R. Danube in this area, a” G. sp. 1″ haplotype was detected. Although introductions by humans cannot be completely excluded, this species group has low economic importance and is unlikely to be translocated as stock. Therefore, we believe that the presence of” G. sp.1″ is more likely due to the effects of recent or paleohydrological changes. Since large areas of the Bánát region have constantly or intermittently been flooded in the geological recent past, the downstream sections of the Temes-Béga systems did not separate from each other clearly [46]. In previous centuries, an extensive canal system for flood protection and navigation has been established in this area; moreover, the lower reaches of the rivers have also been regulated. The recent Danubian estuary of the River Temes at Pancsova is also the result of contemporary water management interventions. Additionally, the two rivers are connected by canals on their middle and lower sections (coordinates: 45.760677, 21.803850; 45.746708, 21.602980; 45.363144, 20.534530). Consequently, these rivers can be considered as an interconnected water system by now. Presumably, the hydrogeological circumstances of this area are responsible for us finding that the gudgeons collected in these three rivers did not show considerable phylogenetic differences. On the other hand, they slightly separated from the stocks collected from northern areas. This slight isolation is confirmed by the emergence of the hitherto unknown haplotypes H5 and H6. At the sites located at the Körös drainages (Sites 1 and 2) and two Maros tributaries (Sites 8 and 9), only” G. sp. 1″ haplotypes were detected. However, unlike the Béga-Temes system, the already known haplotypes H2 and H3 were observed almost exclusively in these sites. In accordance with our previous study, 82% of the studied samples collected from the Middle-Tisza watershed showed these two haplotypes, therefore, they seem to be commonly distributed in the R. Tisza basin [37].

Contrary to its strong hydrographic separation, the commonly-distributed haplotypes H2 and H3 could be detected in the River Olt (Site 10) as well. Although deliberate introductions by humans cannot be completely excluded in this case either, this finding can also be explained by paleohydrological changes. Specifically, the River Olt lost its contact with the Maros water system at the end of the Middle Pleistocene [47, 48]. Consequently, the fish fauna inhabiting these river systems might also have been connected. This assumption is reinforced by the fact that the Petényi barbel (*Barbus petenyi* Heckel, 1852) occurs in both river systems [49]; moreover, other aquatic groups such as amphipod crustaceans show a similar phylogenetic pattern in the Maros and Olt drainage [50].

Notwithstanding the detected high level of genetic diversity (nine haplotypes in a relatively small area), we have to consider that these phylogenetic differences are relatively small. For instance, in our study, the largest indicated interspecies genetic difference (*G. obtusirostris* vs. *G. gobio*) hardly exceeded 3%. Phylogenetic analyses (Fig. 2) separated the valid species with low probabilities. The primary reason for this phenomenon is the relatively low intraspecies genetic distances, but the degree of segregation is further reduced by the presence of taxonomically unclear groups (SW or” Balcanic clade”, and „G. sp. 1″). However, both the Network analysis and the nucleotide difference computations showed that „G. sp.1″ occupies an almost equidistant position between the two valid species (*G. obtusirostris*, and *G. gobio*). Moreover, this haplogroup is located on a branch of the network, therefore it seems to be a slightly differentiated but phylogenetically distinct entity.

Spatial distribution of the newly revealed and the literature data reinforced the assumption that the range of *G. gobio* also extends to the middle and lower watershed of the River Danube, but only with sporadic occurrence. Additionally, our results suggest that the distribution of *G. carpathicus* is much narrower than it is presumed, and is limited only to the northern Carpathian area. The taxonomically not clarified cryptic species” G. sp. 1″ was shown to be widely distributed in the Southeastern area of the Carpathian basin as well. Additionally, the geographic distribution and co-ocurrence data on” G. sp. 1″ reinforced the suggestion of Zangl et al. 2020 [38] that the co-existence of different Gobio lineages enables their hybridisation in the Middle Danubian region. A mitochondrial DNA analysis, due to its limitations, cannot prove this hypothesis; therefore it is beyond the scope of our present study. Further genetic investigations are required to analyze the possibilty of hybridisation.

## Conclusions

Despite the relatively limited geographic range of the study, our results provide important information helping us to re-evaluate the phylogenetic, taxonomic and distribution features of Central European gudgeons. In light of the new results, we refuted the assumption that the Carpathian stream-dwelling gudgeons could be characterized by a genetic continuum. In fact, in both the middle and lower part of the Tisza drainage system, a slightly separated, but phylogenetically distinct and generally distributed, undescribed gudgeon species can be found. Moreover, this group was detected in the surrounding watercourses (R. Olt and Temes) that have a current hydrologic connection to the Tisza drainage, or had one in the recent past. In order to clarify the taxonomic position of the previously unknown group, additional (e.g. morphological) investigations are required.

Our findings together with the results of other phylogenetic studies suggest that sampling in the Carpathian aquatic system can provide valuable additional genetic information. Therefore, we conclude that the increased spatial resolution of sampling in this area may also help to clarify the phylogenetic relationships of other problematic taxa.

## Methods

### Sampled rivers

In our study the sample sites were designated to be representative regarding the entire water system of the Southeast Carpathian area. The sampled river sections were situated in Romania, and they flow directly or indirectly into the R. Tisza or into the R. Danube [46, 48]. Since the sampled rivers have no English names, both their Hungarian and Romanian names are indicated here. The Sebes-Körös/Crisul Repede, Fehér-Körös/Crisul Alb collect the waters of the Apuseni Mountains and flows into the River Tisza. The Kis-Küküllő/Târnava Mică and Sztrigy/Strei are the tributaries of the River Maros/Mures, which is one of the longest tributaries of the River Tisza. The rivers Béga/Bega, Temes/Timiș and its tributary, the River Berzava/Bârzava, as well as the River Krassó/Caraș and Néra/Nera, collects their waters from the Bánát and the Southern Carpathian regions. The River Béga/Bega is the Southest tributary of the R. Tisza, while the other four rivers flow into the R. Danube. The Olt/Olt and Argyas/Argeș rivers flow also into the Danube, but outside of the Carpathian basin. While the River Olt breaks through the Southern Carpathian Mountains to the South, the R. Argyas/Argeș originates on the Southern slope of the Southern Carpathians. Both rivers reach the Danube on the area of the Wallachian Plain (see: Fig. 1, and Table 1.). To clarify presentation, only the Hungarian names of the rivers are shown.

### Field collections, sampling

Southeast Carpathian stream-dwelling gudgeons (*Gobio* sp. *n* = 56) were collected from the above-mentioned 11 rivers. Two to six individuals were collected per site by electrofishing in the autumn of 2016 and 2018 (Fig. 1, and Table 1.) Fishing licences/authorization for scientific purposes of the ANPA - Agentia Nationala pentru Pescuit si Acvacultura: 08/21.03.2016 and 08/26.03.2018. Fin clip samples were obtained for phylogenetic investigations and stored in 96% ethanol at − 20 °C until DNA extraction. After fin tissue sampling, fish were released to the river section where they were collected.

### Genetic methods

DNA was extracted from 10 to 20 mg of fin tissue using DNeasy Blood and Tissue kit (Qiagen, Germany) according to the manufacturer’s instructions. Quality and quantity of the extracted DNA were measured using a NanoDrop 2000c Spectrophotometer (Thermo Scientific, USA). DNA of the 56 samples was used for the amplification of the mitochondrial control region (mtCR). Although the mitochondrial sequence analysis has its own limitation - as it is not useable for hybridisation studies- but in this work we wanted to reveal the phylogenetic features of the sampled gudgeon stocks. Moreover the mtCR was the most widely employed locus in previous studies dealing with the *Gobio* stocks living in this area [17, 37, 38], therefore this locus was the best suited for our goals. A 711 bp sequence of mtCR was amplified by polymerase chain reaction (PCR) using the primers CR159 (5′-CCCAAAGCAAGTACTAACGTC-3′) and CR851 (5′-TGCGATGGCTAACTCATAC-3′) [17]. PCR was carried out in a final volume of 40 μL containing 0.02 U/μL Phusion Hot Start II DNA Polymerase, 5X Phusion Green HF Buffer (Thermo Fisher Scientific), 200 μM dNTPs (Thermo Fisher Scientific), 500–500 nM primers and 200 ng template DNA. Reaction was performed in a GeneAmp 9700 PCR System (Applied Biosystems) using the following conditions: 98 °C for 1 min, followed by 35 cycles of 98 °C for 10 s, annealing at 60 °C for 30 s, and an extension at 72 °C for 25 s, with a final extension at 72 °C for 5 min. PCR products were purified using QIAquick Gel Extraction Kit. PCR products were sequenced with BigDye Terminator v3.1 Cycle Sequencing Kit, using ProFlex Thermal Cycler and ABI 3500 Genetic Analyser (Applied Biosystems) using POPO7 polymer and 50 cm capillary array according the recommendation of the manufacturer. Sequences were trimmed manually using FinchTV 1.4.0 (Geospiza) then the 612 bp mitochondrial sequences were aligned to using MUSCLE [51], as implemented in MEGA X [52]. Calculation of sequence polymorphism and haplotype detachment was performed using the FaBox online software [53]. The obtained haplotypes were compared with the ones uploaded to the GenBank using MegaBlastN online software [54]. In order to reveal the taxonomic relationships, alignment of all haplotypes found in this study and haplotypes of other gudgeon species described from the adjacent regions (e.g. Central Europe, Balkan Peninsula, and Anatolia) was performed. The GenBank codes of these haplotypes are indicated in Table 2.

**Table 2:** GenBank haplotypes used for the Bayesian tree and Network computation

N^o^	*Gobio obtusirostris* Valenciennes, 1842	“SW haplogroup”	“G. sp.1”	*Gobio gobio* (Linnaeus, 1758)	*G. sp2*	*Gobio skadarensis* Karaman, 1937	*Gobio carpathicus* Vladykov 1925	*Gobio ohridanus* Karaman, 1924	*Gobio insuyanus* Ladiges, 1960
1	KC757328	KC757330	KC757340	EU131542	EU131551	EU131568	EU131559	EU131572	EU131576
2	KC757329	KC757331	KC757342	EU131543		EU131569	EU131552	EU131570	EU131574
3		KC757332	KC757339^a^	EU131544		EU131567	EU131561	EU131571	EU131579
4		KC757333	KC757341^a^	EU131545			EU131560	EU131573	EU131580
5		KC757334		EU131546					
6		KC757335							
7		KC757336							
8		KC757337							
9		KC757338							

Number (N^o^) of haplotypes correspond with the numbers displayed at each species/haplogroups in Figs. 2 and 3. ^a^ haplotypes are not shown because they are identical with the H2 nad H3 haplotypes

The phylogenetic relationships of the newly revealed haplotypes and literature data was revealed by a Markov chain Monte Carlo method (B/MCMC), and was performed using MrBayes 3.2.7 [55]. implemented into the NGPhylogeny.fr online tool [56]. A Bayesian tree was constructed using the best fitting GTR evolutionary model approach with 10^6^ generations, 100 sampling frequency, and a burnin of 10,000 samples. The posterior probability values are indicated at the supported nodes. The Bayesian tree was built using a *Romanogobio vladykovi* (Fang, 1943) haplotype (GenBank acc. Number: MK975878) as an outgroup sequence. Haplotype network from the newly detected haplotypes and literature data was constructed using median-joining algorithm in Network v. 10.0.0.0 [57] software. Similar haplotypes were classified arbitrarily into haplogroups (see “enframings” in Fig. 3).

## Data Availability

The datasets supporting the conclusions of this article are available in the GenBank repository [https://www.ncbi.nlm.nih.gov/]. The newly indicated haplotypes have been deposited in the GenBank under accession N^o^s. MT547546–52 and MT967499–500.

## Abbreviations

A: Area
mtCR: Mitochondrial Control Region
G.: Gobio
“G. sp. 1” and” G. sp. 2″: A taxonomically doubtful *Gobio* haplogroups, firstly mentioned by Mendel et al. (2008)
L: Length
R.: River
